# An Electromagnetic Sensor for the Autonomous Running of Visually Impaired and Blind Athletes (Part II: The Wearable Device)

**DOI:** 10.3390/s17020381

**Published:** 2017-02-16

**Authors:** Marco Pieralisi, Valentina Di Mattia, Valerio Petrini, Alfredo De Leo, Giovanni Manfredi, Paola Russo, Lorenzo Scalise, Graziano Cerri

**Affiliations:** 1 Department of Information Engineering, Universita’ Politecnica delle Marche, 60121 Ancona, Italy; marco.pieralisi@univpm.it (M.P.); v.petrini@univpm.it (V.P.); a.deleo@univpm.it (A.D.L.); g.manfredi@univpm.it (G.M.); paola.russo@univpm.it (P.R.); g.cerri@univpm.it (G.C.); 2 Department of Industrial Engineering and Mathematical Science, Universita’ Politecnica delle Marche, 60121 Ancona, Italy; l.scalise@univpm.it

**Keywords:** electronic travel aids, visually impaired athletes, wearable device, virtual running lane

## Abstract

Currently, the availability of technology developed to increase the autonomy of visually impaired athletes during sports is limited. The research proposed in this paper (Part I and Part II) focuses on the realization of an electromagnetic system that can guide a blind runner along a race track without the need for a sighted guide. In general, the system is composed of a transmitting unit (widely described in Part I) and a receiving unit, whose components and main features are described in this paper. Special attention is paid to the definition of an electromagnetic model able to faithfully represent the physical mechanisms of interaction between the two units, as well as between the receiving magnetic sensor and the body of the user wearing the device. This theoretical approach allows for an estimation of the signals to be detected, and guides the design of a suitable signal processing board. This technology has been realized, patented, and tested with a blind volunteer with successful results and this paper presents interesting suggestions for further improvements.

## 1. Introduction

The promotion of sports and physical activity is a key factor in the improvement of the quality of one’s own health and social life, and is especially true with people affected by physical disabilities. It is known that playing sports can help them face and overcome their physical limitations and increase their self-esteem and independency, thus providing them with opportunities to keep healthy and meet new people.

Despite efforts during the last few years to design smart electronic travel aids to support the mobility of people affected by visual disabilities [1,2,3], only a few concern the possibility of increased autonomy during sport [4,5,6]. This is surprising, because although some sports do not require special aids or guides, many others—such as running—require the presence of a sighted guide during the activity, significantly limiting the autonomy and performance of blind athletes.

In this context, we have recently demonstrated the actual possibility of using electromagnetic (EM) technology to design smart systems to help a blind subject walk or run autonomously [5]. In Part I of this paper, our research focused on fixed infrastructure which focused on equipping running tracks with a virtual and invisible hallway where blind athletes could safely run without the need for a sighted guide. In brief, the variable currents flowing through two wires (the transmitters) were placed directly on the ground perimeter of a standard athletic track (400 m length), generating two magnetic fields that can be detected by a coil (the receiver) worn by the runner. Therefore, the overall system is composed of a transmitting unit—whose main features were reported in Part I of this paper—and a receiving unit, whose design and realization will be discussed in this paper.

It is worth noting that when designing a device to be worn during sport or in general physical activity, it is necessary to account for specific requirements such as comfort, weight, and ease of use. This is especially important when athletes are affected by visual disabilities. The results of a recent user-focused assessment conducted among visually impaired and blind volunteers [7] highlighted the requirements for electronic travel aids where the main issues were related to the physical characteristics and appearance of the devices. Users wanted devices which were unobtrusive, inconspicuous, and easy to carry. Blind people preferred discrete devices that were not eye-catching and alienating, but small, light-weight, and preferably consisted of a single unit. Moreover, many users considered hands-free operation to be extremely useful, and provided different modes of outputs (e.g., tactile or auditory).

Currently, most wearable magnetic sensors proposed in the literature or available in the market [8,9] can satisfy the main requirements listed above, especially in terms of reduced weight and dimensions. However, in order to provide these characteristics, it is necessary to work at high frequencies (beyond the MHz range). This is a critical point, because—as explained in Part I of this paper [10]—for the running-track system, the frequency f = 100 kHz was chosen as a trade-off working frequency because it satisfied important system requirements, but was also high enough to induce a sufficient voltage in the receiving sensor whilst still being able to provide a uniform current along the whole cable.

Since none of the existing sensors were suitable as magnetic sensors for the running-track system, an ad hoc wearable device has been designed and realized. Our design is a flat loop composed of 40 turns, and can be worn as a belt. The two magnetic fields generated by the transmitting unit induce different electromotive forces in the flat loop sensor. Basically, the signal processing unit connected to the sensor calculates the difference between the two voltages, and allows information about the position of the user inside the lane to be delimited by the two wires. If the value of the difference overcomes a certain threshold, it means that the athlete is getting too close to one of the borderlines and the unit generates a vibrational signal to warn the user. It must be emphasized that the proposed device was designed for athletes in training; and the present system is not applicable for road marathons, since two long wires need to be placed along the entire marathon path. An alternative electromagnetic system [11] has been proposed for these types of events, or other innovative GPS-based devices could be investigated.

The paper is divided as follows: In Section 2, an overview of the theoretical considerations for designing the receiving magnetic sensor is provided; in Section 3, the receiving unit and all its features are described; and in Section 4, the first tests carried out in collaboration with a blind runner are shown and discussed. A final discussion and some conclusions will be reported in Section 5. An appendix at the end of the paper will provide the mathematical details concerning the theoretical model proposed in Section 2.

## 2. System Overview: Theoretical Considerations

In this section, the theoretical model used to characterize and optimize the main parameters of the receiving system is presented.

**Figure 1:**
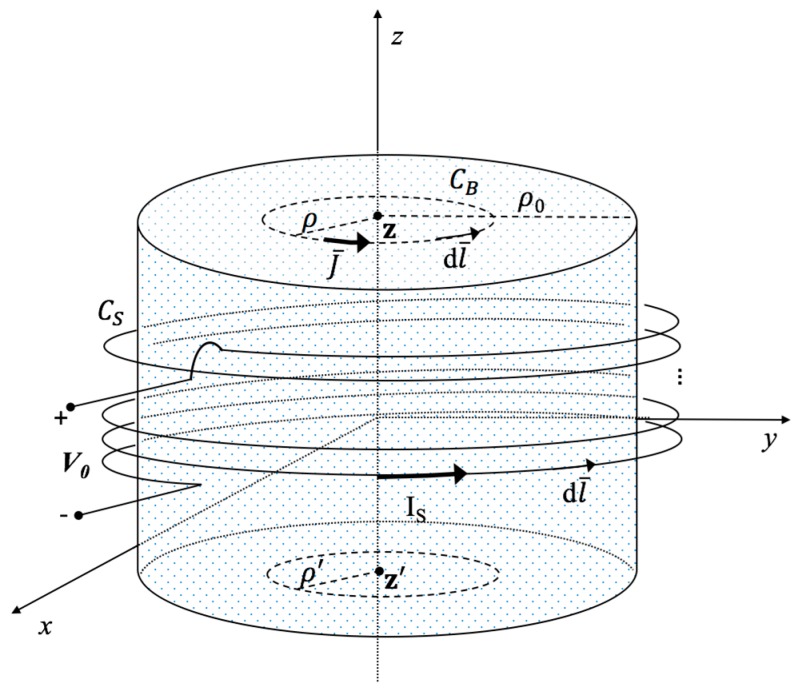
Schematic representation of the sensor coil wrapped around the body, represented by the cylindrical structure.

To develop the analytical model, the following assumptions were considered: (1) that the external magnetic field is uniform in the worn sensor; (2) that the human body is schematically represented by a homogeneous cylinder of lossy material having a radius ρ_0_ and height 2h. Figure 1 depicts the geometry of the problem.

The receiving sensor is a belt composed of a loop of N turns that is wearable by the user, whose task is to detect the magnetic fields generated by the two wires of the transmitting giant loop lying on the ground. To calculate the voltage induced into the receiving coil by the magnetic fields, a theoretical approach has been developed that also considers the mutual coupling between the body and the receiving loop.

**Figure 2:**
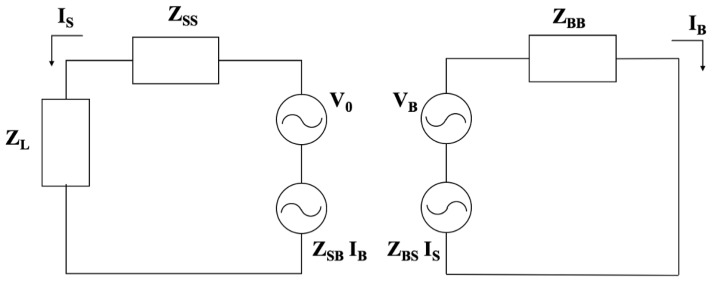
The equivalent circuit used for the electrical representation of the body–sensor coupling where Z_SB_ = Z_BS_ is the mutual-coupling between the sensor and the human body; Z_SS_ and Z_BB_ are the impedances of the sensor and of the human body respectively.

Figure 2 shows a circuit representation in terms of the Z-matrix parameters of the electric problem: the circuit on the left-hand side refers to the sensor (quantities denoted with subscript S), while the one on the right-hand side is a lumped circuit model of a generalized distributed network modeling the body (quantities denoted with subscript B).

By applying Faraday’s law, the equations governing the body–sensor interaction to determine the Z-matrix parameters are achieved.

(1) $$V_{0}-Z_{W}I_{S}=j\omega \oint_{S_{W}}\vec{A}\left(I_{S}\right)\cdot d\vec{l}+j\omega \oint_{S_{W}}\vec{A}\left(\vec{J}\right)\cdot d\vec{l}$$

(2) $$\oint_{C_{B}}\frac{\vec{J}\left(\rho ,z\right)}{\sigma_{B}}\cdot d\vec{l}=-j\omega \int_{S_{B}}{\vec{B}}_{0}\cdot d\vec{S}-j\omega \oint_{C_{B}}\vec{A}\left(\vec{J}\right)\cdot d\vec{l}-j\omega \oint_{C_{B}}\vec{A}\left(I_{S}\right)\cdot d\vec{l}$$

Equation (1) pertains to the coil, and the line integrals are calculated along a path *S_W_* over the wire surface. $Z_{W}=\frac{Nr}{\sigma a\delta }$ is the sensor wire ohmic impedance (*a* being the wire radius, *σ* the copper conductivity, and *δ* the penetration depth); and $\vec{A}\left(I_{S}\right)$ and $\vec{A}\left(\vec{J}\right)$ are the magnetic vector potentials due to the sensor current I_S_ and body current density $\vec{J}$, respectively.

Equation (2) pertains to the body. The line integrals are calculated along a generic circular path *C_B_*, and the surface integral represents the flux of the external field *B*_0_ through the surface *S_B_*, lying on a plane parallel to the x–y plane, and enclosed by *C_B_*; *σ_B_* is the body conductivity.

The contribution to the voltage induced in the sensor is given by the external field ${\vec{B}}_{0}=\mu_{0}{\vec{H}}_{0}$, which is generated by the wires lying on the ground (length *L* = 400 m). Furthermore, we considered that only the z-component of the field (perpendicular to the ground) was sensed by the wearable coil; and this field can be expressed as per Reference [12].

(3) $$H_{0_{z}}=\frac{1}{8\pi^{2}}\underset{L}{\int }I\left(x^{\prime },y^{\prime }\right)\overset{\infty }{\underset{-\infty }{\iint }}\left[\zeta \left(d\bar{l}\cdot \hat{y}\right)-\eta \left(d\bar{l}\cdot \hat{x}\right)\right]\;\frac{2e^{-j\gamma_{1}z}}{\left(\gamma_{1}+\gamma_{2}\right)}\;e^{j\left[\zeta \left(y-y^{\prime }\right)+\eta \left(x-x^{\prime }\right)\right]}d\zeta d\eta$$

(4) $$\gamma_{1}=\sqrt{k_{1}^{2}-\left(\varsigma^{2}+\eta^{2}\right);\;}\gamma_{2}=\sqrt{k_{2}^{2}-\left(\varsigma^{2}+\eta^{2}\right)}$$

where *k*_1_ and *k*_2_ are wavenumbers for free space and ground, respectively; ζ and η are the spectral integration variables used for the Green’s function representation. In Equation (3), the assumption of a circular track of radius R with a total length equal to the one of a real stadium has been adopted. The detailed derivation of the Z-matrix parameters is reported in the Appendix.

The motivation beyond such an analytical approach was twofold: the evaluation of the coupling between the sensor and the body and the possibility to have a priori characterization of the sensor. This information allowed for better design and optimized sensors.

Table 1 summarizes the main physical parameters of the first sensor prototype realized. These results have been used to calculate the impedance of the sensor and the coupling with the body. Table 2 summarizes the values obtained expressed as impedance, resistance, and inductance.

Looking at Table 2, it can be seen that (1) the value of Z_BS_ is small, meaning that the mutual coupling between the body and the sensor is not significant and can be neglected; and (2) the term Z_BB_ introduces only a resistance contribution, due to the ohmic losses related to the currents flowing into the tissues. The information about the negligible value of Z_BS_ is an important result, as it implies that the wearable sensor can be designed independently of the body, avoiding a customized calibration procedure for different athletes.

To validate the results obtained by the analytical model, a preliminary campaign of measurements was carried out. Table 3 shows a comparison between the values of resistance (*R*) and inductance (*L*) measured for three different situations: (1) belt wrapped around an empty human phantom; (2) belt wrapped around a human phantom filled with salty water; and (3) belt wrapped around a human body. Measurements were referred to the input port of the sensor. As predicted by the theoretical model, the main evidence indicates that the presence of the body wrapped by the coil does not produce a great variation in the parameters of the sensor.

Against such a background, the development of a theoretical model has given us an a priori indication about the impedance of the sensor in order to optimize the receiving system during the design.

## 3. Receiving Unit

**Figure 3:**
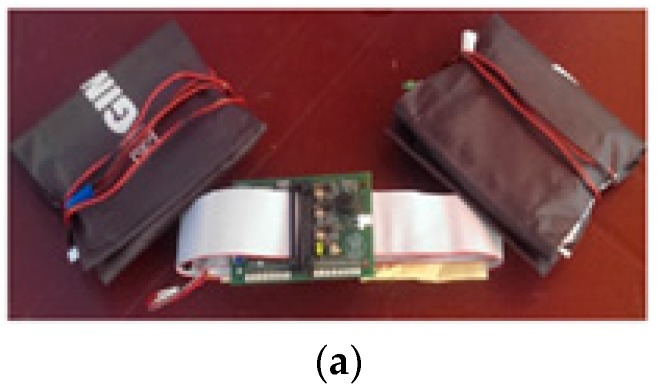

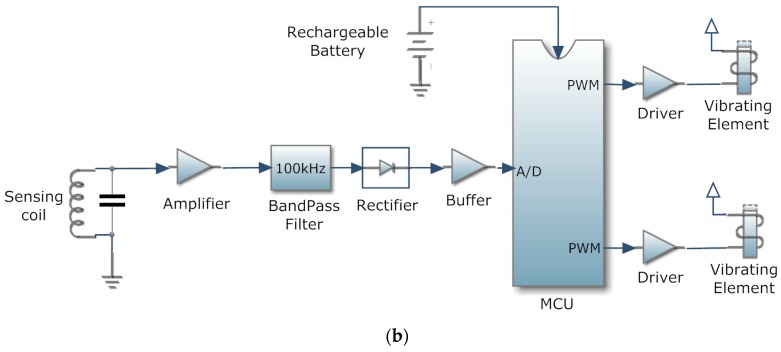
(**a**) A picture of the receiving system; (**b**) Block diagram of the receiving circuit: The analog signal detected by the coil is amplified, filtered, and rectified. The microcontroller (MCU) converts the signal into a digital sequence, whose peaks are evaluated and used to drive the vibration motors placed on both arms of the runner. PWM: pulse width modulation.

The following section describes the hardware and software implementation of the receiving unit. As depicted in Figure 3, it consists of a wearable sensor; a customized circuit board for signal acquisition and processing; and two vibrating devices to communicate a warning signal to the athlete when required.

The multi-turn coil that the magnetic sensor is composed of has been realized with a flexible flat cable (i.e., worn as a belt around the hips or sewn onto a t-shirt or trousers) whose ends are connected to the high impedance input of an operational amplifier and to the circuit ground. A discrete capacitor has been added between these terminals to move the overall resonance closer to the working frequency.

**Figure 4:**
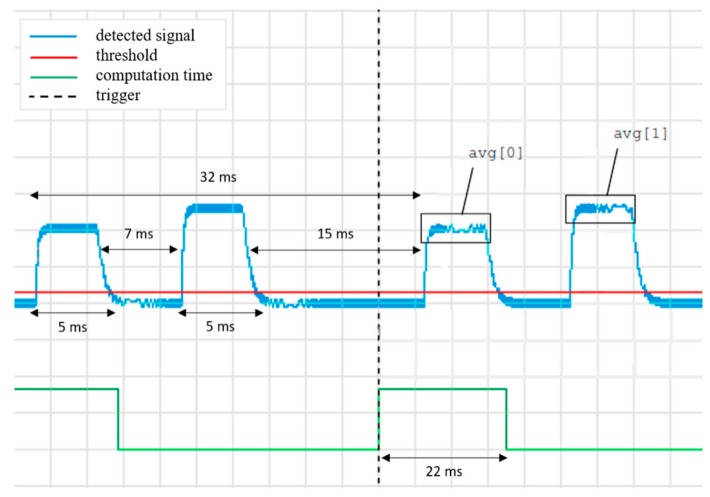
Sample of the analog signal entering the analog-to-digital (AD) converter of the microcontroller (MCU). The two bursts generated by the left and right boundary wires are detected as a pair of consecutive pulses with different amplitudes, according to the runner’s position. The busy flag of the MCU. After 10 ms of signal below the gate threshold (red line), the MCU is triggered to start the evaluation of the sampled buffer. After the computation time, the amplitude difference between the two previously measured pulses is eventually output on the vibration motors.

As thoroughly explained in Part I of this paper, the signal is a sequence of two peaks (maximum induction magnetic field strength B = 24 nT), separated from each other around 7 ms, and repeated every 32 ms. After amplification, the signal enters a second-order band pass filter (Single-Amplifier Biquadratic Active filter [13]), which is then amplified again and rectified. Such an output is then low-pass filtered to reduce the ripples and then buffered again to enter the analog-to-digital (AD) converter of the microcontroller (MCU). The firmware implements a circular buffer 32 ms long with a sampling period $T_{s}=256\;\mu s$ (i.e., 125 samples, corresponding to a sampling rate of about 3.9 kHz). Once the receiver detects no signal for a time longer than $t_{p}=40\cdot T_{s}=10.24\;\mathrm{ms}$, it scans the received buffer backwards trying to identify two consecutive peaks with an intermediate pause no longer than 10 ms. This means that when 40 consecutive samples are below a noise gate level (where the amount is 4% of the range of the AD converter), the whole buffer is evaluated in order to determine the amplitudes of the two peaks which are at an approximate distance of 7 ms. To avoid missed triggers, the computation has to conclude before the next long pause occurs (for example, 22 ms are required to scan the peak heights for a sequence of 125 samples, as shown by the busy flag in Figure 4).

**Figure 5:**
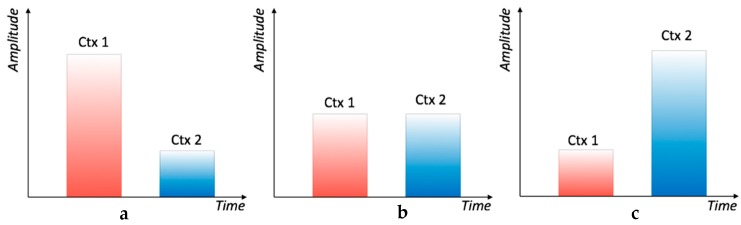
Comparison between the signals: (**a**) athlete position close to the left wire; (**b**) athlete in the central position (secure zone); and (**c**) athlete position close to the right wire.

The peak amplitude average was calculated for both the left and right boundaries, and their difference provided the relative position inside the lane delimited by the two wires, as explained in Part I. Inside the safe invisible hallway, the difference signal had a linear behavior and a maximum electromotive force (emf) value of about 120 mV was measured over a width of 2 m. Considering that the receiver has a sensibility of about 5–10 mV, it can be said that the system was able to confine the user with an accuracy of about 10–20 cm. When the absolute value of such a difference was below a certain threshold, no vibration occurred; however, this threshold is programmable and was set to have no vibration inside the middle lane of the track. As this difference grows—in either a positive or negative direction—a linearly increasing pulse width modulation (PWM) drives the corresponding vibration motor placed on the user’s arms (Figure 5). In brief, the receiving sensor provides feedback about the position of the user inside the safe zone delimited by the two wires, warning the user when they are departing from the middle lane and need to move back towards the center.

The designed working time for a battery pack of 2000 mAh and in the hardest working condition for the system—which requires continuous activity of one of the vibrating actuators—lasts more than six hours, and is more than required for a normal training session.

The information delivered to the user through vibration is updated every 32 ms with a maximum delay of 64 ms in the worst case of sudden variation of position, which at a velocity of 5 m/s (i.e., 18 km/h) corresponds to a distance of about 30 cm.

## 4. Test and Discussion

The EM running-track system reliability and its performance has been tested thanks to the collaboration of an Italian blind athlete, Andrea Cionna, who holds the world record for the fastest marathon run by a totally blind man and has won two bronze medals in blind long-distance running at the Paralympic Games [14].

**Figure 6:**
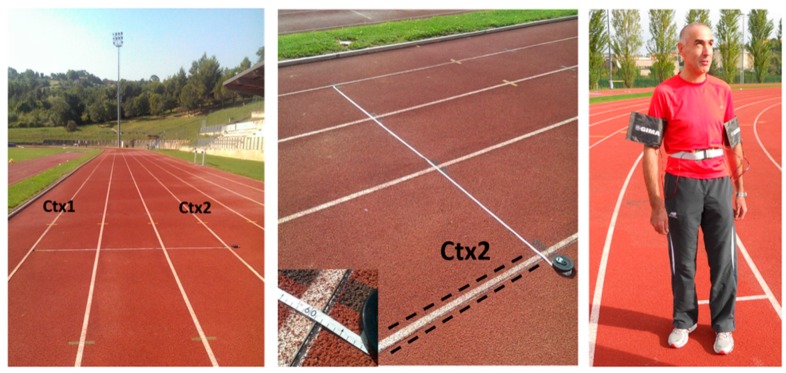
The blind athlete equipped with the electromagnetic sensor at an outdoor running track. Ctx1 and Ctx2 indicate the two wires generating the magnetic field.

The tests were performed at an outdoor running track with a synthetic rubber flooring and eight lanes, with each lane 122 cm wide. Two wires with a length of about 400 m and cross-section of 2.5 mm^2^ were placed on the floor along the whole perimeter of the track, with a distance of approximately 3.6 m from each other, delimiting a path which included the second, third, and fourth lanes of the track. As shown in Figure 6, the athlete was equipped with the receiving system which consisted of a sensor belt around his waist and two vibrational warning signals around his upper arms.

As the volunteer had never used this technology, preliminary training was required to gain confidence in using the system, and in particular with the interpretation of the vibro-tactile warnings coming from both arms. Given that the system is very easy to wear and use, training only took a few minutes, after which the system was tested in two situations of different severity (fast walking and running), the results of which are listed in Table 4.

It was noted that the time required to complete a full lap decreased test by test, while the main velocity increased, confirming that the user grew more confident with the system over time. During normal and fast walking, the athlete maintained a safe and constant pace on the straight course as well as on the curves, faithfully interpreting the vibro-tactile signals coming from his right and left arms, which allowed the user to complete the laps autonomously.

Regarding the running test, the volunteer was able to complete the lap several times, keeping a fast pace both on the straight and curved parts of the track. However, there were times at the end of each curve when the athlete missed the warning signal and consequently departed from the safe zone. According to the blind volunteer, this undesired situation occurred during the tests (indifferently walking or running) as he instinctively tried to receive feedback that gave him the feeling of being in the right position and that the system was working properly. Consequently, he “leaned” closer to the magnetic wall, activating the continuous vibration of one of the sensors on his arms. Therefore, instead of keeping in the central safe zone, he was running on the borderline of the lane, thus from this position, his reaction time to the increasing level of the vibration warning was not sufficiently fast compared to his speed to prevent his exit from the safe zone.

The use of vibrational actuators as the user-interface is in line with the requirement to reduce the over-use of acoustic sensing in the visually impaired athlete. This issue is stringent for any mobility system designed for the visually impaired, and is possibly even more important for runners. If the user prefers systems that leave their arms free, commercially available haptic belts could be used instead of the arm-band system, as the system has been designed to work easily with any user-interface device (haptic belt, voice-recorded systems, etc.).

## 5. Conclusions

In this paper, the design of the receiving subunit of the running-track system proposed in Part I was briefly described. The unit is a wearable device composed of a magnetic belt sensor, a circuit board for signal processing, and two vibrating elements to wrap around the user’s arms.

The idea is to detect and distinguish between the two magnetic fields generated by the wires of the transmitting giant loop lying on the ground in order to guide a blind athlete inside a safe lane. Basically, the receiving magnetic sensor is a loop of N turns, and the theoretical approach developed to calculate the voltage induced by the magnetic fields has been widely described and has accounted for the interaction between the sensors and the body of the user wearing it. Such a theoretical approach has allowed the quantification of the intensity of the received signal to design and realize the circuit board for the signal processing.

A system prototype comprising the transmitting unit described in Part 1 [10] and the receiving unit described in this paper was finally tested thanks to the assistance of a blind volunteer, as we considered that only realistic tests with the end-user were able to uncover any system limitations. All of the tests demonstrated the capability of the sensor to successfully provide feedback about the position of the user inside the safe lane, with warning signals given for impending lane departure. During normal and fast walking testing, the athlete maintained a safe and constant gait, even when approaching curves. In running mode, an improvement of the vibro-tactile interface is needed. The introduction of a continuous and always present low intensity signal to be followed as a guide would allow the athlete’s perception of their own position to increase, thus avoiding the borderlines and any potential departure from the safe zone, as well as their confidence in the correct working of the system.

Important feedback arose from the tests with the end user. The first issue concerned the modality of warning, where it was suggested that rather than receiving no warning when the athlete was inside the safe lane, a continuous and always present low intensity signal would be preferred as a reference signal whose intensity progressively increased as the user approached one of the borderlines. This would allow: (i) an increase of the athlete’s perception of their position; (ii) the improvement of their confidence in the correct working of the system; and (iii) the athlete to avoid being too close to one of the borderlines. The second issue concerned the system response time. This could be solved by optimizing the receiving unit and then reducing such time. Furthermore, the space between the wires could be increased, providing a wider space to the athlete to receive the warning and change direction before reaching the borderline.

Finally, bearing in mind that the system tested was a prototype and that the volunteer had never used this technology before, it can be concluded that the running-track system represents promising technology to support blind athletes during running and training. It is worth noting that the receiving device is simple to use, with only a short training period required; is light weight (about 150 g); and comfortable to wear, so it does not affect athlete performance.

## Figures and Tables

**Table 1:** Main parameters of the sensor.

Radius of the Belt *r* (m)	0.153
Thickness of the belt *t* (m)	5 × 10^−2^
Cu Conductivity *σ* (S/m)	5.80 × 10^7^
Section of the wire *a* (m)	0.15 × 10^−3^
Body Conductivity *σ_B_* (S/m)	0.361

S = Siemens.

**Table 2:** Calculated values of Z-Matrix.

	Z (Ω)	R_s_ (Ω)	L_s_ (μH)
Z_SS_	9.37 + j 534	9.37	850
Z_BS_	j 2.3237	/	3.6982
Z_BB_	44.63 + j 0.0383	44.63	0.0609

**Table 3:** Comparison of R and L values for the sensor wrapped around different materials. All measures were made at 100 kHz and with the same loop geometry.

	R (Ω)	L (μH)
Air	14.1	830
Water	14.6	836
Body	15	840

**Table 4:** Results of fast walking and running tests, performed by a blind athlete on a running track.

	Length (m)	Time	Mean Velocity (m/s)	No. Times out of Safe Zone
Fast walking				
Test 1	400	4′03″	1.64	0
Test 2	400	3′43″	1.79	0
Running				
Test 3	400	3′	2.22	4
Test 4	400	2′45″	2.42	4
Test 5	400	2′32″	2.63	3

## Appendix A

The equivalent circuit components representing the coil can be obtained from Equation (1), by inserting the explicit expressions of the magnetic vector potentials. The current density distribution flowing into the body, assuming cylindrical symmetry, is written as

(A1) $$\vec{J}=J_{0}g\left(\rho ,z\right)\hat{\phi }$$

Consequently, the total current *I_B_* flowing into the body is calculated as the flux of the current density through the body vertical cross-section *C_SB_*:

(A2) $$I_{B}=\int_{CS_{B}}\vec{J}\cdot d\vec{S}=J_{0}\int_{-h}^{h}\int_{0}^{\rho_{0}}g\left(\rho ,z\right)d\rho \;dz=J_{0}F_{N},$$

where *J*_0_ is a constant value and *g*(*ρ,z*) is a function that characterizes the current density distribution in the body. Its flux *F_N_* can be also considered as a normalization factor.

As the current induced into the body is mainly due to the external field ($H_{0}$), uniform throughout the body, a reasonable approximated distribution is

(A3) $$g\left(\rho ,z\right)=\frac{\rho }{\rho_{0}}$$

Hence, the following explicit expressions for the terms of Equation (A1) are obtained:

(A4) $$V_{0}=-j\omega \mu H_{0}\pi r^{2}N$$

(A5) $$Z_{SS}=Z_{W}+j\omega \frac{\mu_{0}r\left(r-a\right)}{4\pi }\int_{-\Phi_{max}}^{\Phi_{max}}\int_{-\Phi_{max}}^{\Phi_{max}}\frac{e^{-j\beta R_{SS}}}{R_{SS}}\cdot \cos\left(\phi -\phi^{\prime }\right)d\phi \;d\phi^{\prime }$$

where:

(A6) $$R_{SS}=\sqrt{r^{2}+{\left(r-a\right)}^{2}-2r\left(r-a\right)\cos\left(\phi -\phi^{\prime }\right)++k^{2}{\left(\phi -\phi^{\prime }\right)}^{2}}$$

*r*: coil radius
*a*: wire radius
Φ*_max_* = 2π N/2
*k* = *t*/(2 Φ*_max_*): helix pitch of the sensor
*t*: sensor thickness

The mutual impedance is

(A7) $$Z_{S_{B}}=j\omega \frac{\mu_{0}r}{4\pi }\int_{-\Phi_{max}}^{\Phi_{max}}\int_{0}^{\rho_{0}}\int_{0}^{2\pi }\int_{-h}^{h}g\left(\rho^{\prime },z^{\prime }\right)\frac{e^{-j\beta R_{S_{B}}}}{R_{S_{B}}}\rho^{\prime }\cdot \cos\left(\phi -\phi^{\prime }\right)d\rho^{\prime }\;d\phi^{\prime }\;dz^{\prime }d\phi$$

(A8) $$R_{S_{B}}=\sqrt{r^{2}+\rho \prime^{2}-2r\rho^{\prime }\cos\left(\phi -\phi^{\prime }\right)+{\left(k\phi -z^{\prime }\right)}^{2}}$$

The same procedure can be applied to Equation (2). However, in this case, the line integral was performed over a generic circular path *C_B_* inside the body. To derive the lumped components of the *Z*-matrix for the body, the equation is multiplied for a weighting function and integrated over the body vertical cross-section *CS_B_*. In our case, the adopted weighting function was:

(A9) $$w\left(\rho ,z\right)=g\left(\rho ,z\right)/F_{N},$$

where the following expressions were achieved

(A10) $$V_{B}=-\;j\omega \mu H_{z\_0}\pi \overset{h}{\underset{-h}{\int }}\overset{\rho_{0}}{\underset{0}{\int }}w\left(\rho ,z\right)\rho^{2}d\rho \;dz$$

(A11) $$Z_{BB}=\frac{1}{F_{N}}\left(\frac{2\pi }{\sigma }\overset{h}{\underset{-h}{\int }}\overset{\rho_{0}}{\underset{0}{\int }}g\left(\rho ,z\right)w\left(\rho ,z\right)\rho d\rho \;dz+j\omega \frac{\mu_{0}}{4\pi }\overset{2\pi }{\underset{0}{\int }}\overset{h}{\underset{-h}{\int }}\overset{\rho_{0}}{\underset{0}{\int }}w\left(\rho ,z\right)\Psi \left(\rho ,\phi ,z\right)\cos\left(\phi -\phi^{\prime }\right)\rho \;d\rho \;d\phi \;dz\right)$$

where

(A12) $$\Psi \left(\rho ,\phi ,z\right)=\overset{2\pi }{\underset{0}{\int }}\overset{h}{\underset{-h}{\int }}\overset{\rho_{0}}{\underset{0}{\int }}g\left(\rho^{\prime },z^{\prime }\right)\frac{e^{-j\beta R_{BB}}}{R_{BB}}\rho^{\prime }d\rho^{\prime }d\phi^{\prime }dz^{\prime }$$

with

(A13) $$R_{BB}=\sqrt{\rho^{2}+\rho \prime^{2}-2\rho \rho^{\prime }\cos\left(\phi -\phi^{\prime }\right)+{\left(z-z^{\prime }\right)}^{2}}$$

It is easy to verify that *Z_BS_* = *Z_SB_* for reciprocity.
